# Assessing Surface Coverage of Aminophenyl Bonding Sites on Diazotised Glassy Carbon Electrodes for Optimised Electrochemical Biosensor Performance

**DOI:** 10.3390/nano11020416

**Published:** 2021-02-06

**Authors:** Zari Tehrani, Hina Yaqub Abbasi, Anitha Devadoss, Jonathan Edward Evans, Owen James Guy

**Affiliations:** 1 Centre for Nano Health, College of Engineering, Swansea University, Swansea SA2 8PP, UK; h.y.abbasi@swansea.ac.uk (H.Y.A.); anitha.devadoss@swansea.ac.uk (A.D.); j.e.evans@swansea.ac.uk (J.E.E.); o.j.guy@swansea.ac.uk (O.J.G.); 2 Department of Chemistry, College of Science, Swansea University, Swansea SA2 8PP, UK

**Keywords:** 4-nitrobenzene diazonium, functionalisation, electrochemical, surface coverage, amyloid-β peptide

## Abstract

Electrochemical biosensors using carbon-based electrodes are being widely developed for the detection of a range of different diseases. Since their sensitivity depends on the surface coverage of bioreceptor moieties, it necessarily depends on the surface coverage of amine precursors. Electrochemical techniques, using ferrocene carboxylic acid as a rapid and cheap assay, were used to assess the surface coverage of amino-phenyl groups attached to the carbon electrode. While the number of electrons transferred in the first step of diazotisation indicated a surface coverage of 8.02 ± 0.2 × l0^−10^ (mol/cm^2^), and those transferred in the second step, a reduction of nitrophenyl to amino-phenyl, indicated an amine surface coverage of 4–5 × l0^−10^ (mol/cm^2^), the number of electrons transferred during attachment of the amine coupling assay compound, ferrocene carboxylic acid, indicated a much lower available amine coverage of only 2.2 × l0^−11^ (mol/cm^2^). Furthermore, the available amine coverage was critically dependent upon the number of cyclic voltammetry cycles used in the reduction, and thus the procedures used in this step influenced the sensitivity of any subsequent sensor. Amine coupling of a carboxyl terminated anti-beta amyloid antibody specific to Aβ(1-42) peptide, a potential marker for Alzheimer’s disease, followed the same pattern of coverage as that observed with ferrocene carboxylic acid, and at optimum amine coverage, the sensitivity of the differential pulse voltammetry sensor was in the range 0–200 ng/mL with the slope of 5.07 µA/ng·mL^−1^ and R^2^ = 0.98.

## 1. Introduction

**Scheme 1:**
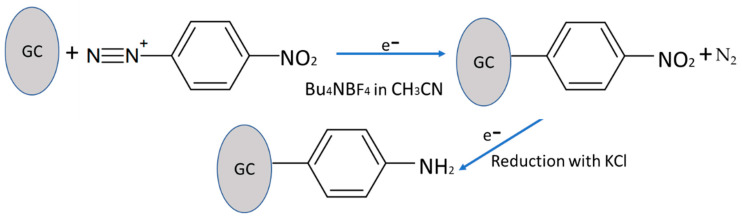
Electrografting of 4-nitrobenzenediazonium tetrafluoroborate salt.

There are several studies that highlight the need for rapid, sensitive techniques and emphasise that biosensor performance is highly dependent on substrate material [1,2]. Carbon-based electrochemical sensor platforms remain a high priority in the biosensor market owing to their low-cost, high sensitivity and simple surface chemistry. This gives great flexibility in designing carbon-based sensors for a wide range of analytes for early disease diagnostics. Various immobilisation strategies have been developed to attach biomolecules to the carbon sensor platform. Here, the selection of the functionalisation process plays a key role in determining the overall sensor performance. Diazonium grafting is one of the most promising methods as it provides a simple technique to immobilise functional groups via covalent attachment [3,4] onto a variety of substrates [5,6]. Diazotisation of carbon electrodes via reducing nitro group to amino group was first reported by M. Delamer et al. in the early 1990′s [7]. Diazonium surface modification and the chemical structure of the modification are shown in Scheme 1.

Later, functionalisation of materials via diazotisation has garnered interest, with several studies dedicated to exploring its potentials in sensing applications [8,9,10,11]. Surface-immobilised groups can induce specific chemical and physicochemical properties to the surface, which may be used in fields, such as chemical [9,12,13] or biological sensing [14,15,16,17,18,19], molecular electronics [20,21,22], microbial fuel cells [11] and energy conversion applications [23,24]. Additionally, diazonium salts have been used for the attachment of metal (aluminium, gold, etc.) nanoparticles [25,26,27], oxide nanoparticles [28,29,30] and nanotextured anti-icing surfaces [31].

Quantifying the efficiency of surface modification is critical to achieving the best sensor performance. A wide range of tools has been reported to determine the efficiency of the diazonium grafting process and quantify the surface coverage of nitrophenyl groups produced. For example, in 1995, Y. C. Liu et al. used high sensitivity surface Raman spectroscopy to obtain spectra from the monolayers of nitrophenyl groups covalently bonded to glassy carbon (GC) and highly ordered pyrolytic graphite (HOPG). They demonstrated that the electrochemical reduction of 4-nitrophenyl diazonium ions in acetonitrile resulted in the formation of 4-nitrophenyl radicals, which, in turn, covalently bonded to the glassy carbon surface. In their work, cyclic voltammetry, X-ray photoelectron spectroscopy (XPS) and Raman spectroscopy data were used to estimate the surface coverage of 4-nitrophenyl as 6.5 × l0^−10^ mol/cm^2^ and 1.6 × l0^−10^ mol/cm^2^ on glassy carbon and HOPG, respectively. Surface coverages from the reduction of diazonium salts for different methods and substrates are given in Table 1.

Antibody immobilisation plays a critical role in determining the immunosensor performance.

**Scheme 2:**
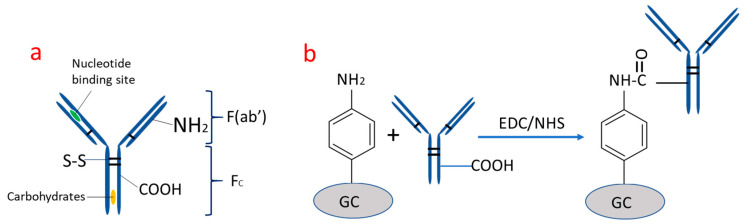
(**a**) Antibody important chemical species for targeting, (**b**) EDC/NHS coupling of the antibody carboxyl group to surface amine group.

Antibodies are composed of hundreds of amino acids to form the characteristic Y-shape, where the carboxyl (–COOH) group is positioned at the lower end of this Y-shape structure (Fc region- = —Scheme 2a). Through the two upper end parts of this Y-shape, which are amine-terminated (Fab region), each antibody is able to bind two antigen species. The orientation, chemical species for targeting, dimensions of antibodies all influence the attachment of the functional groups, such as -NH_2_ and –COOH, to the substrate.

Traditionally, the carboxyl group of the support surface is activated by carbodiimide and reacted with amino groups of antibodies to result in their cross-linking. This will lead to the random orientation of the antibody [37,38]. Therefore, ethyl-3-(3-(dimethylamino)-propyl) carbodiimide hydrochloride (EDC)/ N-hydroxysuccinimide (NHS) (EDC/NHS) coupling of antibody amine and carboxyl groups to surface carboxyl and amine groups [37] was selected to ensure correct orientation of antibody (Scheme 2b).

Alternatively, activating the carboxylic acid (Fc region) of the antibody and attaching them onto the amine-terminated support surface would improve the antibody orientation [39].

Additionally, our recent work already demonstrated that improving and optimising the amine surface coverage onto the support system (carbon, graphene, etc) would improve the sensor performance by improving the antibody immobilisation via carbodiimide reaction [17,40,41,42]. In addition, the utilisation of carboxylic acid groups of antibodies for binding also prevents the potential loss of biological activity of the antibody fragment.

Our primary motivation for this work was to establish a clear understanding of the influence of diazonium functionalisation efficiency over the biosensor performance. Our interest lies in developing electrochemical biomedical sensors made by reduction of electrode bound nitrophenol groups to amines, followed by reaction of these amine groups with sensor molecules, such as antibody receptors. A key factor in determining sensor sensitivity is the surface coverage of receptor molecules, and this, in turn, depends on the surface coverage of the intermediate aminophenyl groups. Despite extensive research on diazotisation of carbon-based substrates, previous studies have not quantified the electrochemical conversion of 4-nitrophenyl groups to aminophenyl groups [33,34]. This research aimed to combat exactly these problems; we provided not only the optimum conditions but also a rapid technique that is effective. Our focus was to report the quantification of the electrochemical conversion of 4-nitrophenyl groups to aminophenyl groups, which, to the best of our knowledge, has never been reported. The effect of the number of CV scans was also discussed in detail. Furthermore, in order to prove this argument, we attached the antibodies on diazotised surfaces of all different scan rates.

In this work, we explored this issue by quantifying the amine group coverage via measurement of electron transfer during electrochemical addition of ferrocene carboxylic acid (FCA) to aminophenyl groups on glassy carbon and highly ordered pyrolytic graphite (HOPG) electrodes. Here, we also investigated the effect of nitro to amino CV reduction scans by controlling the number of scans. The efficiency of our experimental parameter optimisation was studied by developing biosensors prepared by coupling between the electrode bound phenylamino and a carboxyl-terminated anti-beta amyloid antibody specific to Aβ (1-42) peptide, a potential marker for Alzheimer’s disease. Here, Aβ (1-42) was used as a model biomarker to illustrate the successful diazotisation and validate our claim of achieving diazotisation via optimisation of the experimental parameters. Aβ (1-42) is a promising biomarker for Alzheimer’s disease detection, and our group has also previously worked with it and as such made an active decision to use it as the biomarker for supporting better amine coverage in this work.

## 2. Materials and Methods

Materials 4-nitrobenzenediazonium tetrafluoroborate 97% (4-NPD), tetrabutylammonium tetrafluoroborate 99% (BU_4_NBF_4_), 1-ethyl-3-(3-(dimethylamino)-propyl) carbodiimide hydrochloride (EDC), N-hydroxysuccinimide (NHS), ferrocene carboxylic acid (FCA), potassium chloride, acetonitrile (ACN) and phosphate-buffered saline tablets (PBS; pH 7.4) were all purchased from Sigma Aldrich (St. Louis, MO, USA) and used as received. All reagents were of analytical grade, and the deionised water was used for the cleaning of the glassy carbon electrodes. Highly ordered pyrolytic graphite (HOPG) was purchased from MikroMasc (Wetzlar, Germany) with the following specifications: grade ZYA, mosaic spread with the value of 0.4° ± 0.1°, double-sided and with thickness 1 mm and the size of 10 × 10 mm^2^.

Biomarker amyloid-beta peptides Aβ ((1-42) (ab120301)) and anti-beta amyloid antibody (ab224275), specific to Aβ (1-42) peptide Aβ, were purchased from Abcam (Cambridge, UK).

Amyloid-beta (1-42) was supplied in the lyophilised form by Abcam (Cambridge, UK). It was then dissolved in 10 mM sodium hydroxide, followed by gentle Vortex for less than 1 min to make a homogeneous solution. The peptide stock solution was then aliquoted and stored at −20 °C. Under these highly alkaline conditions, the peptide is fully dissolved and exists only as monomers [43].

### 2.1. Electrochemical Measurements

Electrochemical experiments were carried out on an Autolab electrochemical workstation (PGSTAT 302N Metrohm, Utrecht, The Netherlands) at room temperature using a conventional three-electrode system: a bare or modified glassy carbon electrode (GCE) (MF-2012, 3 mm diameter, BASI, West Lafayette, IN, USA) was used as the working electrode, with a platinum wire counter electrode and Ag/AgCl (3 M NaCl, + 0.197 V vs Standard Hydrogen Electrode (SHE)) reference electrode, and a scan rate of 50 mV/s. The GCEs were polished stepwise with aqueous alumina slurries of 0.3 and 0.05 μm particle size using micro-cloth, followed by rinsing thoroughly with isopropyl alcohol (IPA) and then with deionised water for 30 min.

Electrochemical grafting of the diazonium salt was performed using 0.1 M BU_4_NBF_4_ electrolyte in acetonitrile. Typically, blank cyclic voltammetry (CV) was recorded using a freshly cleaned GCE in the electrolyte, following which, the active diazonium compound (5 mM) was added to the electrolyte, and further voltammograms were recorded for 5 cycles.

Subsequent electrochemical reduction of the nitro group (NO_2_) to an amino group (NH_2_) was carried out in 0.1 M KCl in water:ethanol (9:1) electrolyte, using reduction cyclic scanning at 50 mV/s scan rate between −1.2 V and + 1 V vs Ag/AgCl.

Functionalising the amine groups with FCA was carried out as follows: 5 mM EDC/NHS (ratio 1:1) solution was added to 250 µM FCA solution, followed by 40 min of incubation at room temperature. The resultant solution was drop-cast onto the freshly prepared amine-terminated GC and left for 10 min before scanning between −0.3 V and 0.3 V at 5 mV/s in fresh PBS solution, which acted as the electrolyte.

Diazonium functionalisation to prepare a sensor incorporating a carboxyl terminated anti-beta amyloid antibody specific to Aβ (1-42) peptide was carried out using an EDC/NHS protocol [3,22,30]. The Aβ antibody was “activated” in 5 mM EDC/NHS solution for 40 min. This was followed by drop-casting the solution over the electrode surface and incubating for 30 min at 4 °C. The electrode was then rinsed thoroughly with deionised water and dried with N_2_. After that, amyloid-beta (Aβ (1-42)) peptides were added to the electrode surface ranging from 0 ng/mL to 200 ng/mL at 4 °C, each for an incubation time of 20 min. Differential pulse voltammograms (DPV), obtained by sweeping the potential from −0.4 V to +0.3 V with a step potential of 5 mV, an amplitude of 25 mV and interval time of 0.5 s, were used in examining the attachment of antigens to antibody.

### 2.2. Characterisation Techniques

The layers that form the electrodes were characterised to determine their thickness and topography using different techniques to check the quality of the functionalisation of the layers. These techniques included atomic force microscopy (AFM) and scanning electron microscopy (SEM). These were applied to the different stages of functionalisation.

Atomic force microscopy (AFM) was carried out using a JPK NanoWizard II (Berlin, Germany), in intermittent contact mode with tip resonant frequency, spring constant and nominal radius of 320 kHz, 40 N/m and 8 nm, respectively.

Scanning electron microscopy (SEM; Ultra-High-Resolution FE-SEM S-4800, Hitachi, Tokyo, Japan) was carried out at 10 kV acceleration voltage and a 20 mA emission current. The magnification was approximately × 3 k and working distance 10 mm.

## 3. Results and Discussion

### 3.1. Surface Functionalisation of Glassy Carbon via Diazotisation

**Figure 1:**
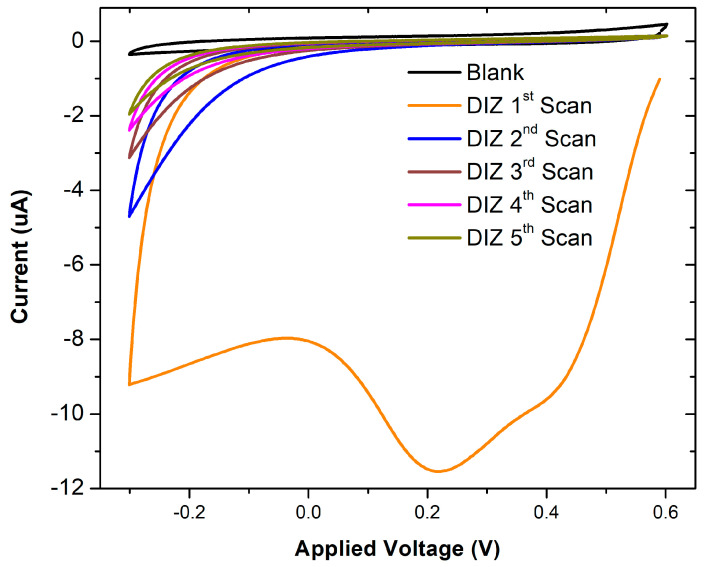
Cyclic voltammograms (50 mV/s) of glassy carbon electrode (GCE) in Bu_4_NBF_4_ in acetonitrile 0.1 M before (black curve) and after the addition of diazonium (DIZ) (orange curve); after repeated voltammetry cycles (five scans, fifth scan olive green curve), there was no further reduction. The experiment was repeated four times.

Figure 1 shows the cyclic voltammetry response of GCEs before and after the addition of diazonium to the electrolyte. As anticipated, no redox processes were noticed for the blank scan (black line), and with the diazonium salt present, a broad irreversible peak (orange curve) was observed at 0.2 V (vs Ag/AgCl), which corresponded to electron transfer associated with the cleavage of dinitrogen from the diazonium salt. This peak completely disappeared in subsequent scans, indicating no further reduction, presumably due to saturation of the available GCE surface with nitrophenyl groups (olive green curve). This observation was consistent with previous reports [44].

### 3.2. Electrochemical Reduction of 4-Nitrophenyl Layers on Glassy Carbon

**Scheme 3:**
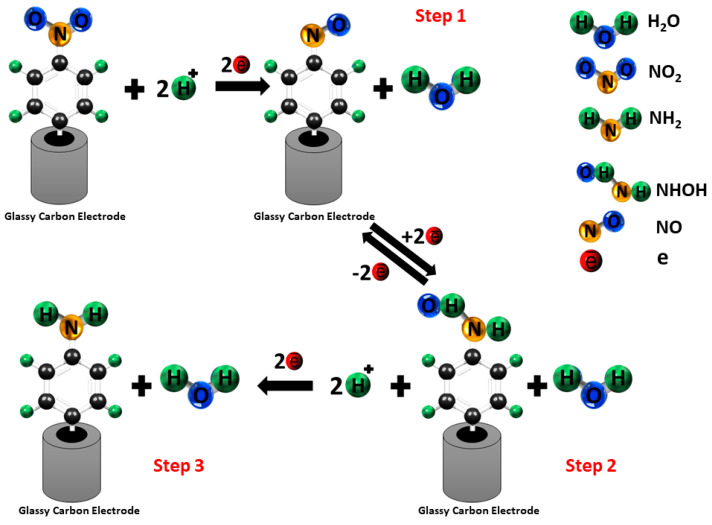
Electrochemical reduction pathway of the nitro group (NO_2_) to an amino group (NH_2_) in an aqueous electrolyte.

Electrochemical reduction of the nitro group (NO_2_) to an amino group (NH_2_) reduction occurs in three steps, as shown in Scheme 3 [19].

**Figure 2:**
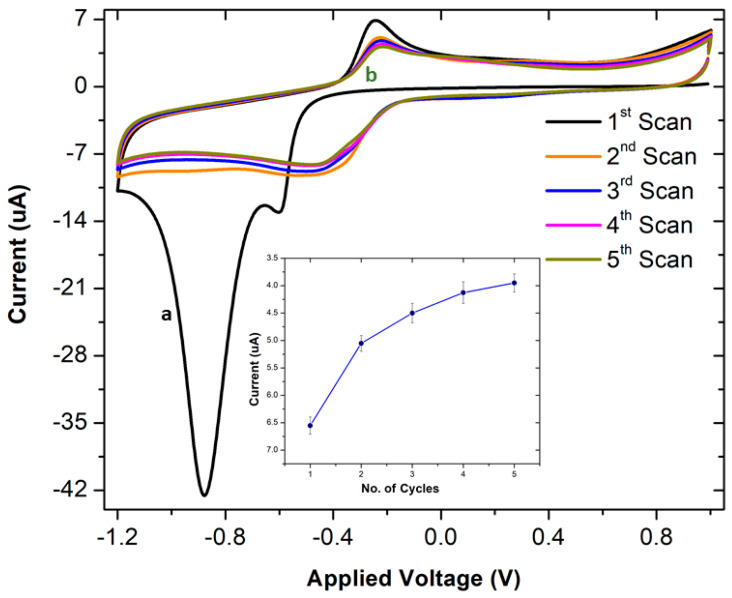
Cyclic voltammograms (50 mV/s) of the reduction of surface-grafted 4-nitrophenyl on GCE in 0.1 M KCl in water:ethanol (9:1) electrolyte: (**a**) black curve—one scan; (**b)** green curve—five scans. The inset figure shows the change of peak current with the number of cycles at −0.2 V. The experiment was repeated four times.

Figure 2 shows the cyclic voltammetry response of 4-nitrophenyl-modified GCEs upon subsequent reduction cycles. A strong irreversible reduction peak was observed at −0.9 V at the first scan corresponding to the formation of the hydroxylamine (PhNHOH). In the subsequent scans, a pair of reversible redox peaks disappeared at a half-wave potential of approximately (−0.45 V). This reversible electrochemistry was attributed to the formation of the hydroxy aminophenyl on the GCE surface. The CV data indicated that the reduction of Ar-NO_2_ was irreversible.

It was also observed from Figure 2 that three CV scans were sufficient for aminophenyl conversion, as overlap in the reduction peaks indicated that no further reduction occurred on the electrode surface after three scans (Figure 2 inset).

### 3.3. Quantification of Surface Amine Groups

**Scheme 4:**
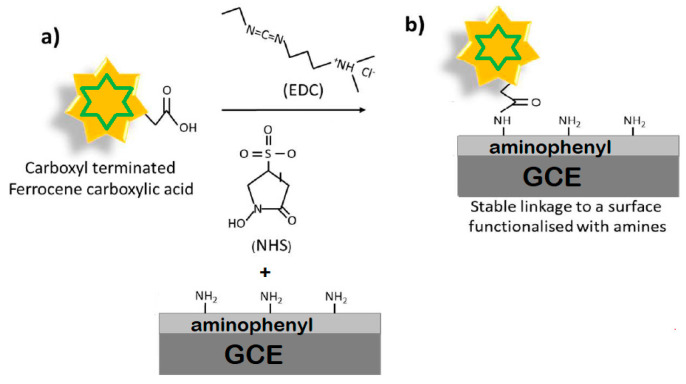
Immobilisation mechanism of electrochemically active ferrocene carboxylic acid (FCA) on to an amine-modified glassy carbon electrode (GCE) through carbodiimide linking.

The surface coverage of amines on the modified electrode was quantified by functionalising the amine groups with FCA, using a standard N-(3-dimethylaminopropyl)-N’-ethyl carbodiimide EDC/NHS coupling reaction (Scheme 4), as follows.

Carboxyl-to-amine crosslinking used the carbodiimide EDC and sulfo-NHS. Addition of NHS or Sulfo-NHS to EDC reactions increases efficiency and enables molecules terminated with the carboxylic acid, such as FCA, to be activated for storage and later use. NHS-activation is the basis for synthesising amine-reactive labelling reagents and crosslinking compounds. This mechanism is very well known and reported [44].

**Figure 3:**
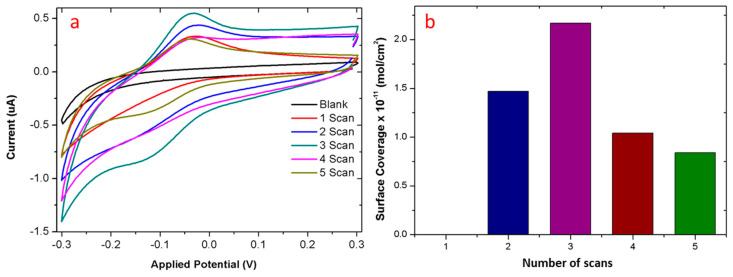
(**a**) Cyclic voltammograms (5 mV/s) of ferrocene carboxylic acid (FCA)-functionalisation of amine-functionalised GCEs prepared using a different number of reduction scans (scans 1–5), in PBS electrolyte. (**b**) The bar chart shows the degree of FCA functionalisation, which is a maximum for the amine-functionalised GCE prepared using three scans.

Figure 3 shows the CV measurements performed in order to confirm the attachment of FCA to surface-bound amino phenyl groups.

The surface coverage of FCA was estimated using Equation (1):

Γ = Q/nFA(1)

where Γ is the surface coverage of FCA (mol/cm^2^), A is the surface area of the electrode (cm^2^), n represents the number of electrons involved in the reaction, F is the Faraday constant (C/mol), Q is the total amount of charge calculated from the integration of the reduction peak recorded using cyclic voltammetry. To calculate the surface coverage of the working electrode, the area under the reduction part of the CV curve can be integrated.

As anticipated, our electrochemical measurements showed no FCA attachment to measurements conducted on the first control of blank GCE due to the lack of amine surface groups on pristine GCE (first control).

It was expected to observe strong FCA binding to the GCE-PhNO_2_ (reduction scan-1) as the current literature reports that the large irreversible reduction peak in the first scan, towards the negative potential, is due to the reduction of p-nitrophenyl to p-aminophenyl. However, in striking contrast, the scan, where GCE-PhNO_2_ (reduction scan-1) underwent only a single reduction cycle in KCl, showed no redox peaks upon FCA functionalisation. However, FCA binding was observed for the electrodes under consecutive scans (reduction scan 2–5 in KCl). The surface coverage of FCA at reduction scan-2 was found to be 14.7 pmol/cm^2^. This might be due to the fact that a single reduction of GCE-PhNO_2_ is not sufficient to generate amine surface moieties, instead produces only an intermediate GCE-PhNHOH moiety, which further needs additional scans to generate amine groups [45].

It was also found that the surface coverage of FCA varied strongly, depending on the number of reduction scans (as shown in Figure 3). It was found that the maximum degree of surface coverage was achieved for the sample prepared at reduction scan-3 (21.7 pmol/cm^2^), whereas any further increase in scan reduced the FCA coverage. The decrease in the surface coverage of FCA in scans 4 and 5 could be attributed to the possible loss of amine groups due to the oxidation. Thus, it is significant to achieve an optimal number of reduction scans (in our case, three scans) to achieve the maximum generation of amino phenyl, beyond which, there is a possibility of losing the generated amines via an oxidation process.

### 3.4. Electrochemical Sensing via Analysis of the Electrodes

In order to confirm this behaviour in a practical sensor, we used diazonium functionalisation to prepare a sensor, incorporating a carboxyl-terminated anti-beta amyloid antibody specific to Aβ (1-42) peptide, a potential marker for Alzheimer’s disease. In this case, the sensor response is a reduction in current in differential pulse voltammetry (DPV) as the bound peptide acts as a barrier to electron transfer at the electrode surface [46,47].

**Figure 4:**
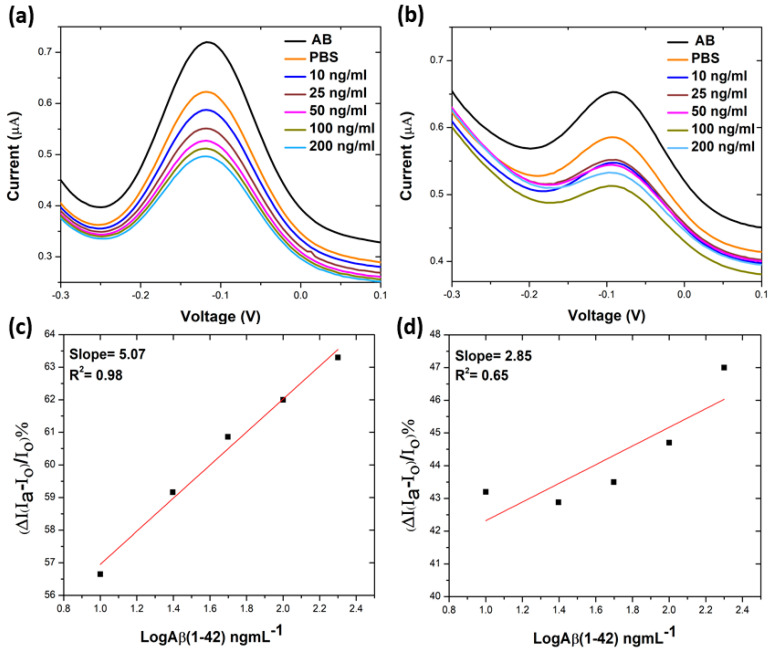
Differential pulse voltammetry (DPV) results for Aβ (1-42) peptide sensors made from amino-functionalised substrates using different numbers of reduction scans. (**a**,**b**) three and five scans; (**c**,**d**) corresponding calibration curves of %IR (I (I_a_ – I_o_) / I_o_) %) (percentage current reduction) against log Aβ(1-42) concentrations.

Figure 4 shows the percentage current reduction (%I_R_) plotted against the log of analyte concentrations. The sensing results indicated that electrode made from an amino substrate prepared using reduction scan-3 showed significantly higher sensitivity (slope: 5.07 and R^2^ = 0.98) than those prepared using scan-5 (slope: 2.85 and R^2^ = 0.65). The regression equation had a logarithmic linear relation with Aβ (1-42) concentration (0 ng/mL to 200 ng/mL), with R² = 0.98 as the correlation coefficient.

**Figure 5:**
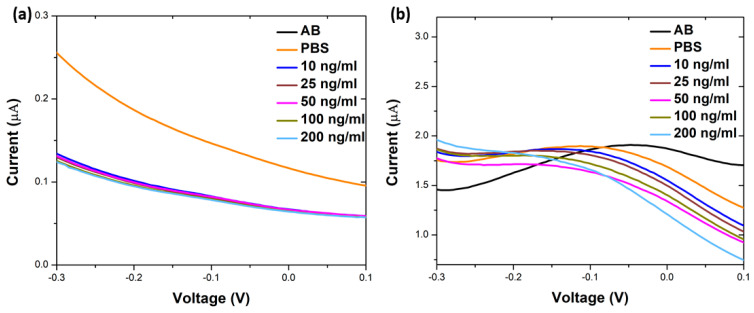
Control experiments pulse voltammetry (DPV) results for Aβ (1-42) peptide sensors made from amino-functionalised substrates using different numbers of reduction scans carried out by (**a**) absence of amine and (**b**) negligible amount of amine are shown.

It is worth mentioning that the control electrodes (GCE, reduction scan-0 and reduction scan-2, Figure 5a,b) showed weak to no significant change in signal upon exposure to Aβ (1-42) peptide. This confirmed that the experimental conditions of diazotisation greatly influenced the sensor performance, and the nitrophenyl to amino phenyl conversion occurred at the reduction scan-2, instead of reduction scan-1.

The pattern of results obtained from these reduction cycles with antibodies matching those conducted with FCA attachment and utilising FCA is a novel, fast and cheap way of gauging the potential sensitivity of a sensor platform made by any particular process, without the need for complete manufacture of the sensor itself.

### 3.5. Characterisation of Surface Morphology Electrode

**Figure 6:**
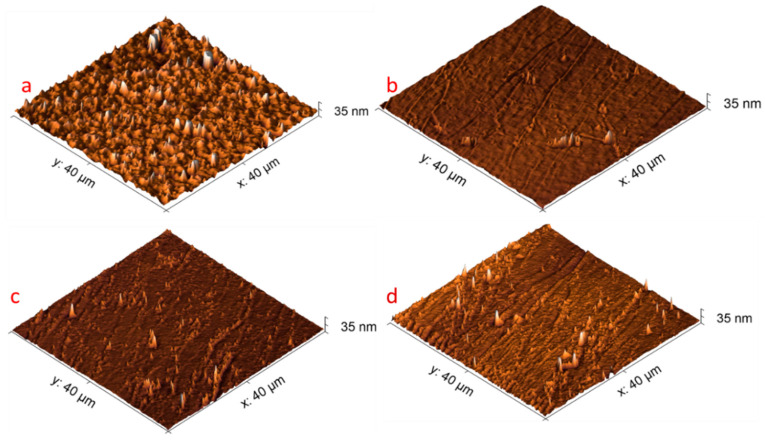
Morphology of highly ordered pyrolytic graphite (HOPG) electrode with atomic force microscopy (AFM). (**a**) blank—before diazotisation, (**b**) after diazotisation—one scan, (**c**) after three scans, (**d**) after five scans.

The effect of surface diazotisation on surface morphology was investigated using AFM on the HOPG electrode. The 40 × 40 µm^2^ AFM scans were conducted in intermittent contact mode. The data were levelled using a linear background, and a high pass filter was used to remove large scale undulations in the HOPG. The roughness and morphology of the functionalised substrate are shown in Figure 6.

The surface roughness appeared similar between the samples after one, three and five scans, with graphite step edges still visible after five cycles. The root mean square (RMS) roughness was generally higher on the blank samples (~5 nm) when compared with the functionalised samples (1–2 nm). However, no clear difference was observed in roughness values between the functionalised samples, with similar topography apparent in the AFM images (Figure 6b–d).

**Figure 7:**
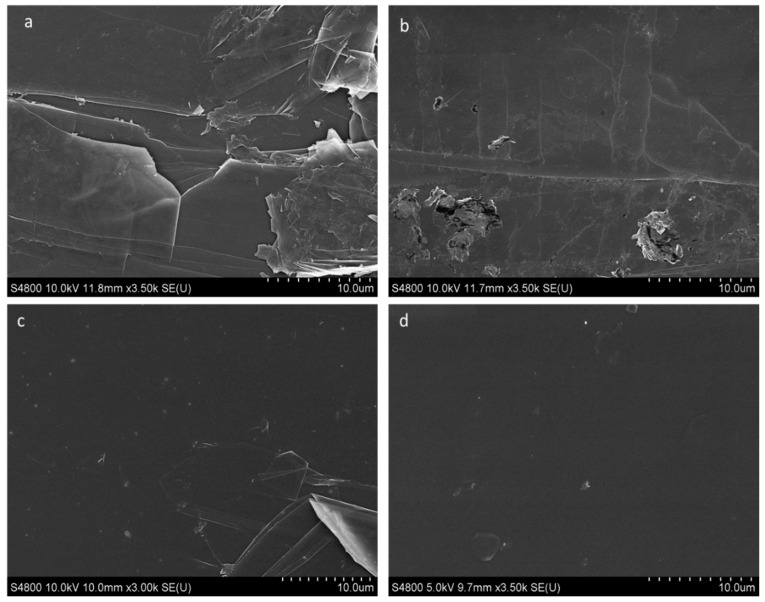
Morphology of HOPG electrode with scanning electron microscopy (SEM). (**a**) blank—before diazotisation, (**b**) after diazotisation—one scan, (**c**) after three scans, (**d**) after five scans.

Scanning electron microscopy (SEM) of the HOPG surface revealed a similar suppression of surface features following initial functionalisation with diazotisation method (Figure 7a,b), in agreement with AFM.

However, a reduction in visible step edges in the underlying HOPG with an increasing number of cycles was also observed (Figure 7b–d). This could be explained by the suppression of low energy secondary electron contrast at step edges due to the presence of an overlayer or thin-film [48].

It is possible that AFM in intermittent contact mode is probing some underlying topography through the functional layer, explaining this discrepancy in observed surface morphology.

## 4. Conclusions

Despite diazotisation being a well-researched topic, there have been no previous reports concerning the quantification of surface coverage of aminophenyl groups, following the conversion of 4-nitrophenyl films (deposited via electrochemical methods), or the optimal conditions required for this reaction. Understanding the extent of conversion of the nitro moiety on different electrode surfaces provides useful information for electroanalytical chemistry researchers in their design of surfaces, and electrochemical procedures, for biosensor molecule immobilisation.

Ferrocene carboxylic acid was introduced as a rapid and cheap assay to assess surface coverage of amino-phenyl groups on electrodes. The number of electrons transferred during the attachment of ferrocene carboxylic acid indicated an available amine coverage of only ca 1/40th, which might be expected from the number of electrons used in the initial diazotisation process, i.e., 2.2 × l0^−11^ mol/cm^2^ compared to 8.02 ± 0.2 × l0^−10^ (mol/cm^2^). Furthermore, the available amine coverage was critically dependent upon the number of CV cycles used in the reduction, and thus the procedures used in this step influenced the sensitivity of any subsequent sensor. The applicability of these ideas was demonstrated via developing a sensor, incorporating a carboxyl-terminated anti-beta amyloid antibody specific to Aβ (1-42) peptide, a potential marker for Alzheimer’s disease. At optimum amine coverage, the sensitivity of the differential pulse voltammetry sensor was in the range 0–200 ng/mL, with a slope of 5.07 µA/ng·mL^−1^ and R^2^ = 0.98. Our work demonstrated an electrochemical-based low-cost surface quantification technique, which could be utilised in combination with other sensor parameters’ optimisation to develop commercially viable, high-performance, carbon-based electrochemical biosensors. Future developments of this work will need to focus on exploring and optimising the sensitivity and selectivity of sensors and using a range of different biomarkers.

## Figures and Tables

**Table 1:** 4-p-nitrophenyl surface coverage on carbon-based substrates.

Substrate	Potential (V)	Surface Coverage (mol/cm^2^) × l0^−10^	Conditions	Ref.
GC		14	4 min electrolysis	[3]
GC		6.5 ± 0.5	10 min electrolysis	[32]
GC		18	4 min electrolysis	[33]
GC		5.6	4 min electrolysis	[34]
GC	−1.06	19 ± 1	t = 10 and 100 min	[35]
SD	−1.17	1.3	N_2_ purged Glove box	[36]
EG		16.6	RT, argon atm. 20 h	[18]
GC	−0.8	22	Argon (oxygen free)	[5]
GC	−0.6	8.02 ± 0.2	Air (atmospheric), 1 min, RT	This work

GC—glassy carbon; SD—single-crystalline diamond surface; EG—epitaxial graphene; RT—room temperature.

## Data Availability

No new data were created or analyzed in this study. Data sharing is not applicable to this article.
